# GNGT1 is a potential prognostic and immunologic biomarker in gastric cancer

**DOI:** 10.1038/s41598-025-08297-4

**Published:** 2025-07-01

**Authors:** Xuchong Huang, Juan Lin, Jian Wang, Weifeng Yang, Wenquan Ou, Xing Huang, Jiahua Chen, Zixing Zhang, Xiaohua Wu

**Affiliations:** 1 Department of Clinical Medicine, Fujian Medical University, Fuzhou, China; 2 Department of General Surgery, Nanping First Hospital Affiliated to Fujian Medical University, Nanping, China; 3 Department of Operating Room, Nanping First Hospital Affiliated to Fujian Medical University, Nanping, China

**Keywords:** G protein subunit gamma transducin 1, Gastric cancer, Biomarker, Immune microenvironment, Bioinformatics analysis, Drug sensitivity, Gastric cancer, Biomarkers

## Abstract

Gastric cancer(GC) is the fifth most common type of cancer worldwide and ranks third in terms of cancer-related mortality. Immunotherapy has shown promising outcomes and greatly extended survival in individuals with advanced stomach cancer. To improve the immunotherapy response in patients with GC, it is necessary to discover new molecular targets. The associations among G protein subunit gamma transducin 1(GNGT1) expression, clinicopathological features, and prognosis were assessed via the UALCAN and Kaplan-Meier databases. The CIBERSORT algorithm in R software and single-sample gene set enrichment analysis(ssGSEA) were used to analyse the proportions of infiltrating immune cells in the high-expression group and the low-expression group.GNGT1 expression was substantially greater in GC tissues than in normal tissues, and patients with GC who had high GNGT1 expression had worse clinicopathological characteristics and survival outcomes. Immunohistochemistry(IHC) experiments on stomach adenocarcinoma(STAD) samples confirmed the aberrant expression of GNGT1 and its association with a poor prognosis. Subsequent investigations revealed substantial negative correlations between GNGT1 and tumour mutational burden(TMB), microsatellite instability(MSI), immune cell infiltration, immune cell gene marker expression and immunological checkpoint expression in patients with STAD.GNGT1 is a reliable biomarker in patients with GC that also has an immunomodulatory function in this disease and may accelerate GC development by suppressing the infiltration of T cells, dendritic cells, M1 macrophages and B cells.

**Supplementary Information:**

The online version contains supplementary material available at 10.1038/s41598-025-08297-4.

## Introduction

The fifth most common kind of cancer worldwide, gastric cancer (GC) ranks third in terms of cancer-related mortality^1^. Because of its low survival rate and high prevalence, GC is a global health concern. Because it is typically detected at a late stage, stomach cancer typically has a poor prognosis, particularly in cases of advanced GC^2^. Radiotherapy, targeted therapy, systemic chemotherapy, and surgical resection have all been demonstrated to be effective therapies for GC^3^. However, their clinical effectiveness against advanced GC is limited. Thus, there is an urgent need for potential therapeutic strategies for patients with advanced GC. Recently, immunotherapy has been recognized as a reliable option for patients with advanced GC, producing favourable results and significantly prolonging survival in patients with advanced GC^4^, which may offer new hope for these patients. Therefore, novel biomarkers linked to immunomodulation are needed to facilitate GC diagnosis, treatment, and prognosis evaluation.

The gamma subunit of transducin, a guanine nucleotide-binding protein present in the rod extracellular segment, is encoded by the G protein subunit gamma transducin 1 (GNGT1) gene. This protein is involved in the RAS pathway and functions as a regulator or transducer in a variety of transmembrane signalling systems^5^. A review of the literature related to GNGT1 suggested that GNGT1 may regulate optic rod photoreceptor function^6^ and that its expression in eye and brain tissues is associated with autoimmune diseases^7^. We are aware of other diseases related to GNGT1, including primary desiccation syndrome^8^ and hypertrophic cardiomyopathy^9^, among others. According to previous research, GNGT1 may help reveal the molecular mechanisms underlying the onset and spread of numerous cancers, including invasive ductal carcinoma of the breast^10^, non-small cell lung cancer^11^, lung adenocarcinoma^12^, rectal cancer^13^, adrenocortical carcinoma^14^ and oesophageal squamous cell carcinoma^15^. In addition, GNGT1 may play a key role in promoting GC resistance to trastuzumab and may be an indicator of the prognosis of GC. Chen et al. screened five genes with good diagnostic value for the diagnosis of GC (GNGT1, KRT7, KRT16, SOX9, and TIMP1)^16^. Other researchers have suggested that GNGT1 is a poor prognostic factor in GC and that high GNGT1 expression is closely linked to shorter patient survival^17^. Nevertheless, the potential role of GNGT1 in GC and how it is related to the tumour immune environment, particularly in terms of immune function regulation, remain unexplored. Recently, immunotherapy, drug sensitivity and immune cell infiltration have been widely studied in the field of tumour genetics. Some studies suggest that grouping patients according to the type of infiltrating immune cells and the expression of immune-related genes may facilitate individualized diagnosis and treatment^18^. Therefore, studying the associations between the proportions of infiltrating immune cells and gene expression in GC patients will provide ideas regarding the personalized diagnosis and treatment of GC. Moreover, drug sensitivity studies may facilitate treatment decision making and improve outcomes.

**Fig. 1:**
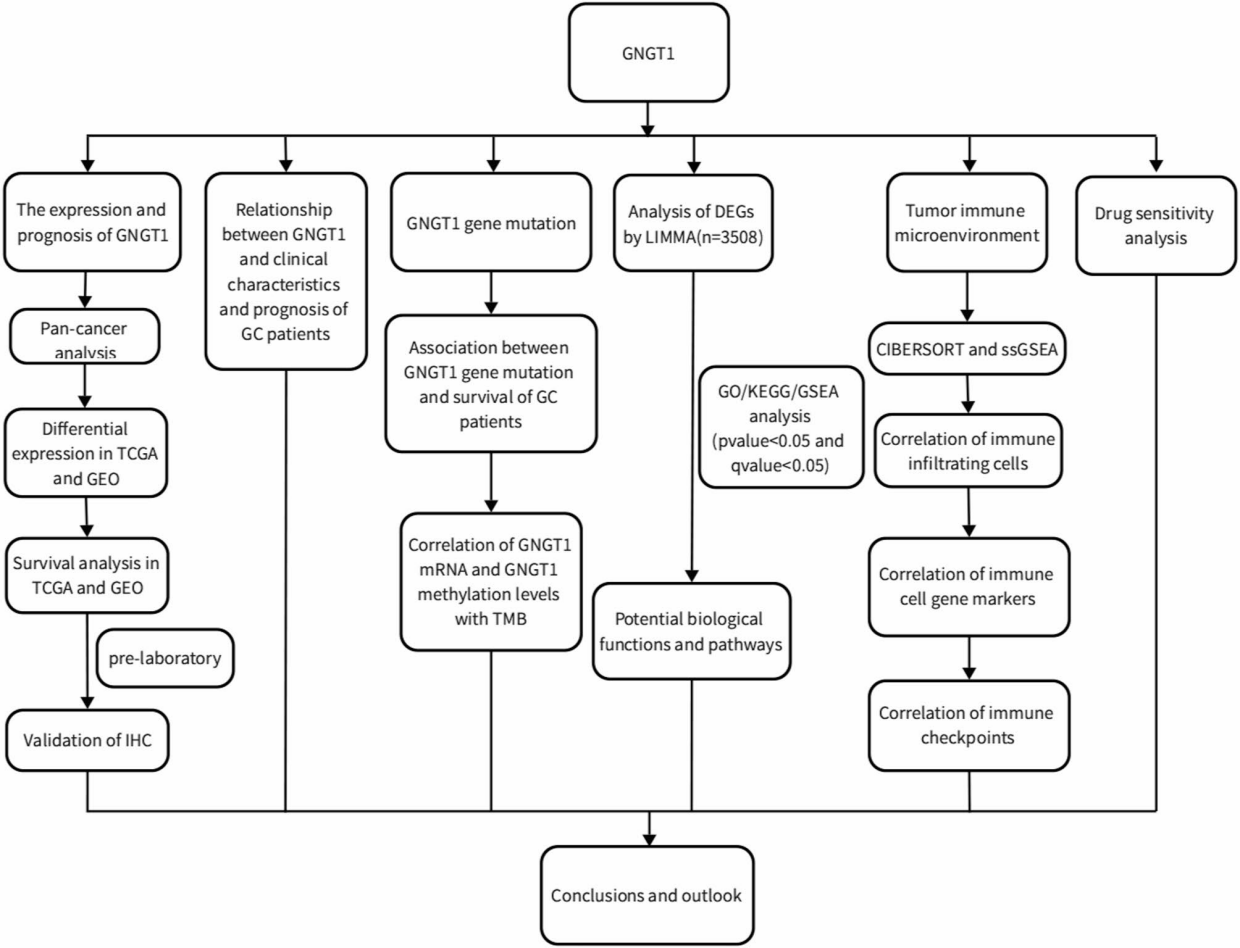
Schematic diagram of the study design.

In our study, we first evaluated GNGT1 expression in GC and its association with prognosis, which was validated with data from a clinical cohort (*N* = 104). Next, we assessed the associations between GNGT1 expression in GC tissues and patient clinical features. To identify the possible biological processes and signalling pathways associated with GNGT1 in GC, we performed GO and KEGG enrichment analyses and GSEA. Multiple databases were used to evaluate the significance of GNGT1 in the GC tumour microenvironment (TME), immune cell infiltration, immune checkpoint expression, immune cell genetic marker expression and immune function. Finally, we performed drug sensitivity analysis. Figure 1 shows the research process.

## Materials and methods

### GNGT1 gene expression

The TIMER2.0 and Sangerbox databases were used to assess GNGT1 mRNA levels across cancers. The TIMER 2.0 database was used to create box plots showing GNGT1 expression in various malignancies^19^. Uniform, standardized pan-cancer data from the TCGA database are included in the SangerBox database^20^. To identify cancers with significant differential expression of GNGT1, we used the above pan-cancer data and signed-rank and unpaired Wilcoxon rank tests. Transcriptomic sequencing and clinical data of GC patients were obtained from the TCGA via the “Bioconductor” software package of R software. We employed the “limma” package of R software to assess the relative expression of GNGT1 in GC and normal tissues.

### Analysis of clinicopathological features

UALCAN was used to assess the relative expression of the target gene GNGT1 in both normal and tumour samples and to assess its correlation with the clinicopathological features of GC patients^21^. The survival data of 1290 GC patients from the TCGA and GEO databases were used to assess the prognostic significance of GNGT1 in GC via the Kaplan–Meier plotter^22^. The GC patients were classified into low- and high-GNGT1 expression groups on the basis of the median GNGT1 level, and differences in their OS and DFS were assessed. The probe ID of GNGT1 was 207166_at.

### GNGT1 gene mutation and its effect on survival

cBioPortal is an open-access tool for interactively examining multidimensional cancer genomic datasets^23^. It allows users to investigate, visualize, and analyse complex cancer genomic and clinical data across multiple dimensions. We applied cBioPortal software to detect GNGT1 gene mutations in GC tissues and analysed the survival of GC patients with and without GNGT1 gene mutations.

### Immunohistochemical validation

We purchased a paraffin-embedded tissue microarray (HStmA180Su20-XT20-011) that contained 180 samples, including 104 GC tissue samples and 76 normal tissue samples, from Shanghai Outdo Biotech Co., Ltd., with 4–6 years of follow-up information available for the corresponding patients. All clinical samples were collected with the informed consent of the patients. The use of the experimental methodology was authorized by the company’s ethics committee (Ethics Code: SHYJS-CP-2001010).

To assess GNGT1 protein levels in GC tissues and adjacent normal gastric tissues, we performed immunohistochemistry. The experimental procedure involved a series of essential steps. Initially, the tissue microarray was processed through baking, dewaxing and antigen repair. The microarray was subsequently incubated with GNGT1 primary antibody (1:3000 dilution, No. bs-13470R, Bioss, Beijing, China) at 4 °C overnight. After exposure to the secondary antibody for 45 min at room temperature, the microarray chip was stained with DAB and then re-stained with haematoxylin. Finally, we used an Aperio ScanScope XT (Leica) to obtain labelled images of the slides. The proportion of positively stained cells was multiplied by the staining intensity of these cells to determine the overall IHC score. The samples were scored according to the percentage of cells with clear positive staining as follows: <5% (0), 5–25% (1+), 26–50% (2+), 51–75% (3+), and 76–100% (4+). The staining intensity was classified into four grades: bright yellow (1+), light brown (2+), brown (3+), and no staining (0). Two experienced pathologists independently evaluated the results. Finally, Sangerbox was used to analyse the score differences between cancerous tissue and adjacent noncancerous tissue samples.

### Functional enrichment analysis of differentially expressed genes (DEGs)

We divided the GC patients from the TCGA cohort into high- and low-GNGT1 expression groups on the basis of the median GNGT1 expression level. We subsequently utilized “DESeq2” software to identify DEGs whose expression levels differed considerably between these two groups. The “DESeq” function in this software reveals genes with statistically significant differences in expression under specific conditions using a negative binomial distribution algorithm. The criteria for DEG identification were a |log2(fold change)|>1 and an adjusted p value < 0.05. The GO and KEGG pathway enrichment analyses of the DEGs were subsequently performed using the R packages “enrichplot”, “ggplot2” and “org.Hs.eg.db“^24^. Next, we performed genome enrichment analysis using the GSEA method in the “clusterProfiler” package in R, with the screening conditions set to |NES|≥1, an FDR < 0.25 and a p value < 0.05. The potential biological functions of GNGT1 were revealed.

### Analysis of the immune microenvironment

In this study, we utilized TIMER2.0 to explore the connections between GNGT1 expression in GC and the proportions of 11 types of tumour-infiltrating immune cells (TIICs), namely, regulatory T cells, CD4+ T cells, CD8+ T cells, B cells, memory B cells, naive B cells, monocytes, myeloid dendritic cells, neutrophils, macrophages, and NK cells. Furthermore, we assessed the correlation between GNGT1 mRNA expression and the expression of immune cell markers. TISIDB, a comprehensive tumour immunology database, was used to assess the associations between GNGT1 expression and immune signatures by integrating multiple data sources, including literature mining and high-throughput data analysis^25^. This allowed us to assess the relationship between the proportions of 24 TIICs in the GC samples and the expression of GNGT1. Next, we used the CIBERSORT algorithm to assess immune cell infiltration in TCGA-STAD tissue samples. The findings of the analysis are displayed graphically. To further explore the relationships between different immune cells, we employed the “corrplot” software package and calculated Pearson correlation coefficients. To evaluate the differences in infiltrating immune cells between the low and high GNGT1 expression groups in GC tissues, we used the “ggboxplot” function of the “ggpubr” software package, supplemented by the “wilcox” function of the “wilcox” software package. In addition, we assessed the relationships between GNGT1 expression and infiltrating immune cells via the “stats” package. Finally, we assessed the connection between GNGT1 and immune function in stomach adenocarcinoma (STAD) by correlating GNGT1 expression with immune function scores via Pearson’s correlation analysis. Immune function scores were also compared between the high- and low-GNGT1 expression groups.

### Immune checkpoint correlation analysis

TIMER 2.0 was used to predict the associations between immunological checkpoint (IC) gene expression and GNGT1 expression. For this analysis, we chose 30 common ICs^26^ that have been documented in the literature. A user-friendly database called XenaShiny makes it simple to explore, analyse, and visualize data from the UCSC Xena database^27^. We explored the associations of GNGT1 with MSI and TMB via XenaShiny and SangerBox. A web-based program called GEPIA2 was used to analyse transcriptome data from the TCGA database in great detail^28^. We used it to investigate the relationships between the proportions of infiltrating immune cells and GNGT1 expression in GC. The expression levels of GNGT1 mRNA in various molecular subtypes were determined via TISIDB.

### Drug susceptibility analysis

Data on the gene expression and drug responsiveness of GC cell lines were obtained from the DepMap database. We divided the cell lines into two groups, the low GNGT1 expression group and the high GNGT1 expression group, on the basis of the median GNGT1 gene expression level. We used log2FC values to assess the sensitivity of the cell lines to a set of drugs. A low log2FC value indicated high drug sensitivity. The R program “pRRophetic” was used to calculate the half-maximal inhibitory concentrations of 138 medications by utilizing dependencies such as “car”, “ridgepreprocessCore”, “genefilter”, and “sva“^29^. Boxplots were created via the “ggplot2” R program.

### Statistical methods

For data analysis, we used a variety of statistical techniques and software, such as Sangerbox (http://vip.sangerbox.com/), the R software (version 4.3.2), UALCAN, TIMER2.0 and the GEPIA database. The data visualization and statistical analysis were primarily performed via R software. To evaluate the statistical significance of differences in GNGT1 immunohistochemistry scores between GC tissues and adjacent normal tissues, we applied Student’s t test. Fisher’s exact test or the chi-square test was used, as appropriate, to assess the associations between GNGT1 mRNA expression and various clinical characteristics of patients with GC. For comparisons between two independent datasets, the Wilcoxon test was applied. To evaluate the connections between the variables, Pearson’s correlation coefficient was computed. The log-rank test was used for survival analysis. A p value of less than 0.05 was considered to indicate statistical significance, and significance levels are denoted as follows: *** for *p* < 0.001, ** for *p* < 0.01 and * for *p* < 0.05.

## Results

### Expression level of GNGT1

**Fig. 2:**
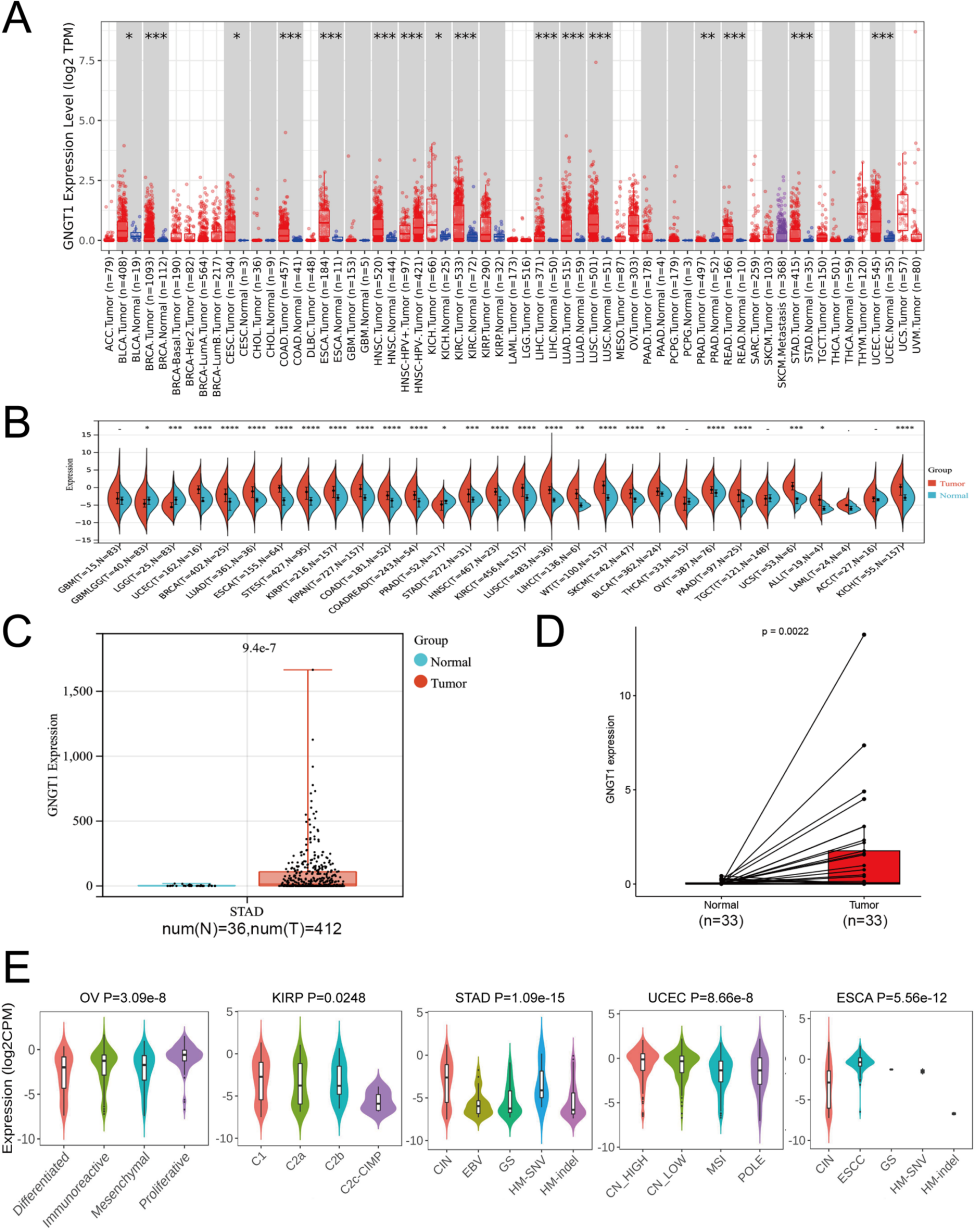
Transcript levels of GNGT1 in human tumours. GNGT1 mRNA expression across cancers from the TIMER 2.0 (**A**) and Sangerbox (**B**) databases. Note: Red and blue indicate tumour and normal samples, respectively. (**C**) Elevated levels of GNGT1 mRNA in GC tissues compared with corresponding normal tissues from TCGA (*p < 0.05). (**D**) GNGT1 mRNA expression in paired tumour and adjacent normal tissues from 33 patients with STAD from the TCGA database. (**E**) GNGT1 expression in different molecular subtypes of cancer was investigated via TISIDB.

First, we used TIMER2.0 (Fig. 2A) and Sangerbox (Fig. 2B) to assess the expression levels of GNGT1 mRNA in different human malignancies. The findings demonstrated that GNGT1 was highly expressed in various cancers, such as STAD, READ, COAD, LUAD, BLCA, BRCA, ESCA, LIHC, LUSC, KICH, KIRP, KIRC, HNSC and UCEC. However, GNGT1 mRNA expression was low in LGG and PRAD. To confirm that GNGT1 is expressed in GC, we assessed RNA-sequencing data from 36 TCGA normal tissues and 412 GC tumour tissues. This analysis revealed that GNGT1 was highly expressed in GC (Fig. 2C,D). Additionally, the different molecular subtypes of STAD, KIRP, OV, ESCA, and UCEC exhibited significant differences in GNGT1 mRNA levels (Fig. 2E).

### The expression of GNGT1 in GC tissues is correlated with clinical parameters

**Fig. 3:**
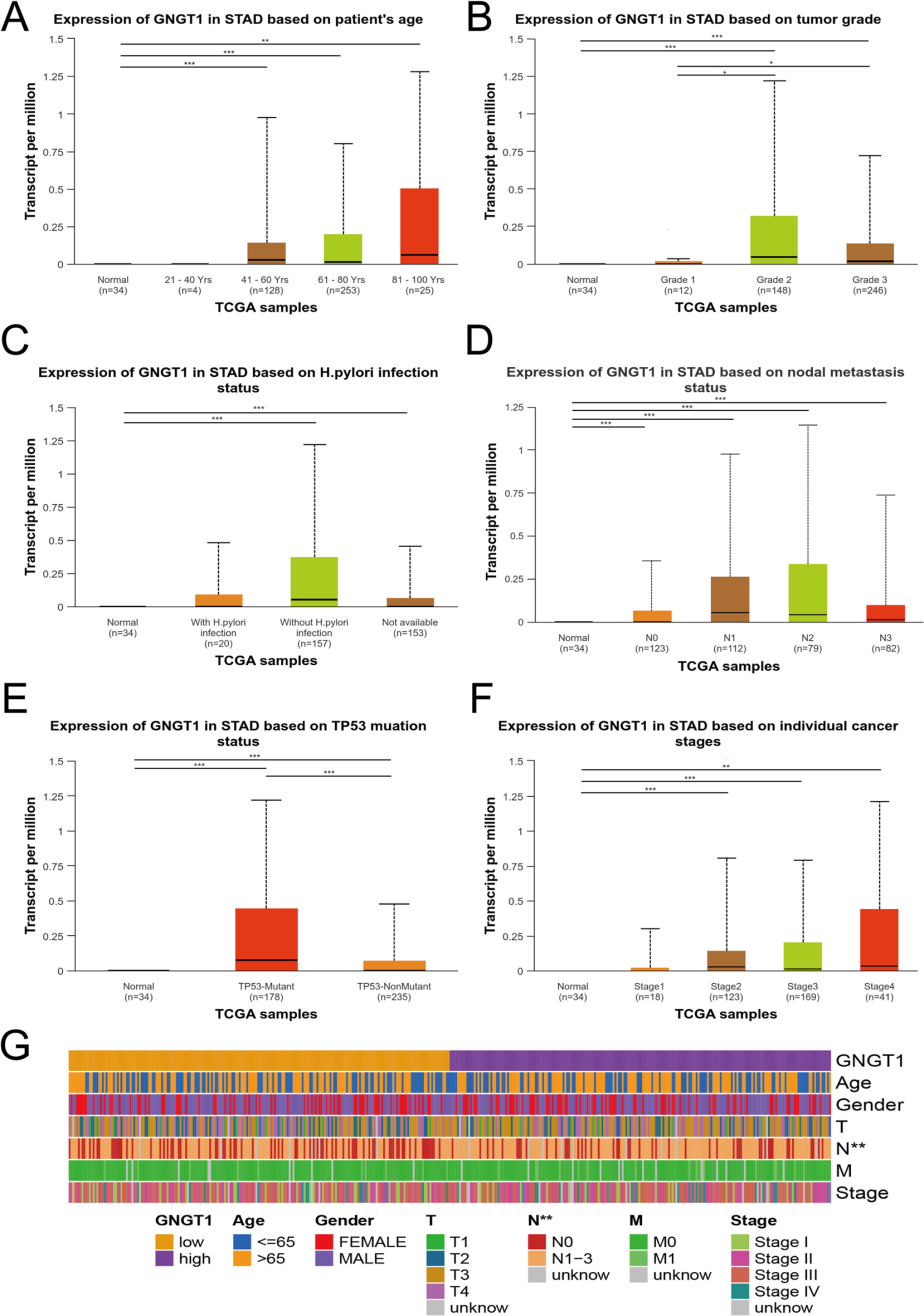
Correlations of GNGT1 expression in GC with clinical parameters. GNGT1 was correlated with the age (**A**), tumour grade (**B**), H. pylori infection status (**C**), lymph node metastasis status (**D**), TP53 mutation status (**E**), and stage (**F**) of GC patients. Heatmap of the correlation of clinical information (**G**).

Using the UALCAN database, the relationships between GNGT1 expression and several clinical characteristics, such as age, grade, TP53 mutation status, H. pylori infection status, lymph node metastasis status and tumour stage, were investigated to clarify the function of GNGT1 in GC. We found that GNGT1 was significantly upregulated in the three age groups GC patients (40–61, 61–80, and 81–100 years old) compared with the normal group, and the 81-100-year-old group presented the highest GNGT1 expression (Fig. 3A). Additionally, GNGT1 expression differed significantly between the grade 1 and grade 2 groups and the grade 1 and grade 3 groups, suggesting that the GNGT1 levels in grade 2 and 3 tumours were much greater than those in grade 1 tumours (Fig. 3B). The expression level of GNGT1 was greater in the H. pylori-infected group than in the uninfected group (Fig. 3C). Additionally, the expression level of GNGT1 was significantly associated with lymph node metastasis, with significant upregulation of GNGT1 in the normal group versus the N0, N1, N2 and N3 groups (Fig. 3D). The level of GNGT1 expression was greater in the TP53-mutant group than in the TP53-nonmutant group and the normal group (Fig. 3E). In terms of tumour stage, GNGT1 mRNA expression was considerably greater in the stage II, III, and IV groups than in the normal group, and the stage IV group presented the highest GNGT1 expression (Fig. 3F). We also generated a heatmap of the correlations with clinical features via R software. This analysis revealed that the proportions of patients with different N stages significantly differed between the low- and high-GNGT1 expression groups (Fig. 3G). These results indicate that high GNGT1 expression may be related to poor clinical features and clinical outcomes.

### GNGT1 expression is associated with the prognosis of GC

**Fig. 4:**
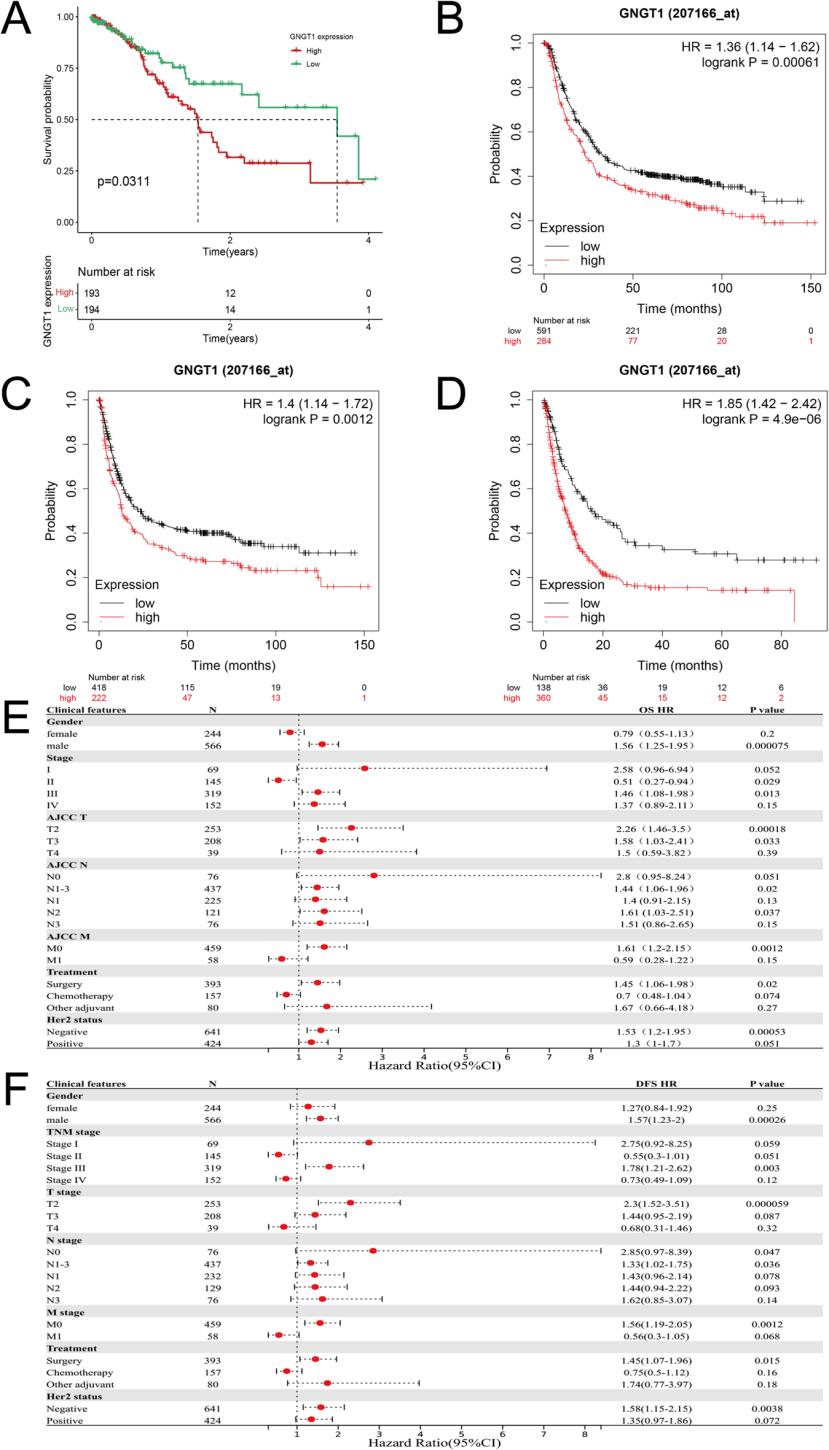
The prognostic significance of GNGT1 in GC. Kaplan–Meier survival curves were generated to evaluate OS (**A**,**B**), PPS (**C**), and DFS (**D**). Additionally, the associations between GNGT1 mRNA expression levels and OS (**E**) and PPS (**F**) were assessed in patients with GC.

We utilized the Kaplan-Meier plotter to assess the prognostic significance of GNGT1 in GC. First, we explored the TCGA-STAD cohort and discovered that patients with GC with low GNGT1 expression had longer OS (*P* = 0.0311, Fig. 4A). To further validate these results, we performed a survival analysis of the GEO cohort, which included 875 GC patients. This analysis yielded similar conclusions, showing that the GC patients with reduced GNGT1 expression had better OS (*P* = 0.00061, Fig. 4B), DFS (*P* = 0.0012, Fig. 4C) and PPS (*P* = 4.9e−06, Fig. 4D). Next, we explored the connection between GNGT1 expression and the prognosis of GC via Kaplan‒Meier plotter. In patients with different clinical characteristics (such as sex, stage III, T2, N1–3, M0, surgical treatment and HER2 status), elevated GNGT1 levels were significantly associated with poorer OS and DFS (Fig. 4E, F). In addition, increased GNGT1 expression was linked to worse OS in patients with stage T3 and N2 GC, as was poorer DFS in those with stage N0 GC. These findings indicate that GNGT1 expression is a potential prognostic marker for GC and is closely related to clinical parameters.

### Association between GNGT1 gene mutation and survival in GC patients

**Fig. 5:**
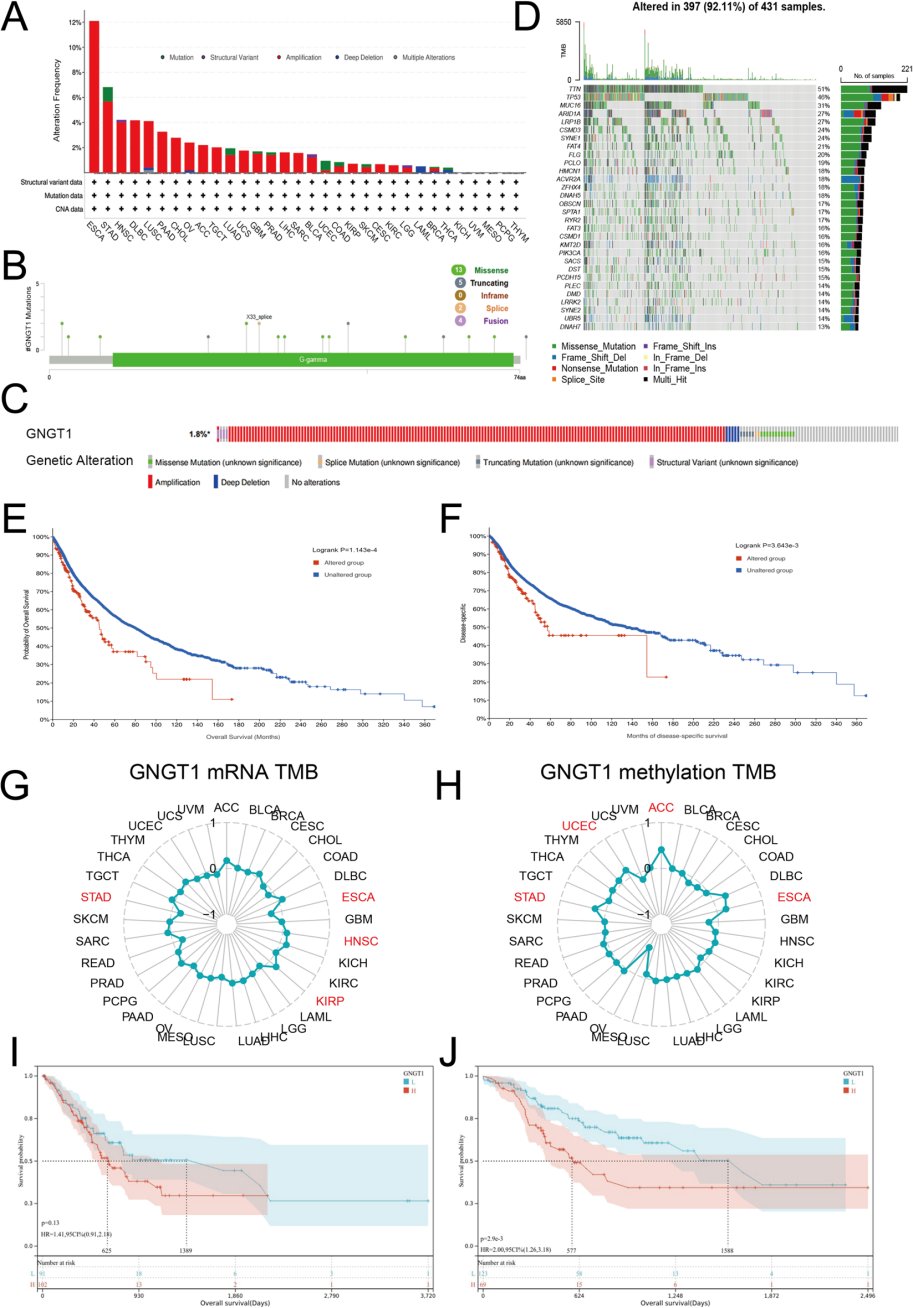
GNGT1 mutation types in GC and survival analysis of patients grouped according to TMB. (**A**) Frequency of GNGT1 gene alterations and mutation types across cancers. (**B**) GNGT1 mutations across protein structural domains. (**C**) Frequency of GNGT1 gene mutations in GC tissues. (**D**) Waterfall plot of GNGT1 in the TCGA-STAD cohort. GNGT1 mutations predict poorer OS (**E**) and DFS (**F**). In GC, GNGT1 expression was negatively correlated with the TMB (**G**), and GNGT1 DNA methylation was positively correlated with the TMB (**H**). A correlation between GNGT1 expression and overall survival was not detected in individuals with GC with a low TMB (**I**) but was detected in individuals with a high TMB (**J**).

Given the importance of gene mutations in tumour growth, we used the TCGA database and cBioPortal software to conduct a thorough investigation of GNGT1 mutations across cancers. The findings indicated that amplification was the predominant GNGT1 mutation, and these mutations were most common in ESCA (12.09%), STAD (6.82%), and HNSC (4.21%) (Fig. 5A). In addition, we identified 24 mutation sites within amino acids 0–74, of which 13 missense mutations, 5 truncation mutations, 2 splice site mutations and 4 fusion mutations were located mainly in the structural domain of the GNGT1 protein. The x33_splice mutation was the most common mutation (Fig. 5B). We further used cBioPortal software to analyse the relationships between GNGT1 gene mutations and GNGT1 expression and survival in GC patients. The results revealed that approximately 1.8% of GC patients presented with genetic alterations in GNGT1, and most GNGT1 mutations resulted in gene amplification in GC (Fig. 5C). GNGT1 mutations correlated well with clinical indicators such as TMB and sex (Table 1). We also generated waterfall plots for GNGT1 in the TCGA-STAD cohort via R software (Fig. 5D). We then investigated the relationship between GC patient survival and GNGT1 mutation status. Compared with individuals without GNGT1 mutations, those with GNGT1 mutations had poorer OS (*P* = 1.143e-4, Fig. 5E) and DFS (*P* = 3.643e-3, Fig. 5F). In addition, we computed the TMB for each sample using XenaShiny and analysed the correlation of the GNGT1 mRNA level with TMB and the correlation of the GNGT1 methylation level with TMB. The findings revealed a negative correlation between the GNGT1 mRNA level and TMB (*r*=-0.197, *P* < 0.0001, Fig. 5G) and a positive correlation between the GNGT1 methylation level and TMB (*r* = 0.237, *P* < 0.0001, Fig. 5H). Among GC patients in the low-TMB subgroup, those with increased GNGT1 expression had shorter OS (*P* = 0.13, Fig. 5I). Similarly, among GC patients in the high-TMB group, those with elevated GNGT1 expression had shorter OS, as indicated by survival analysis (*P* = 0.0029, Fig. 5J).

**Table 1:** Correlations between GNGT1 mutation status and clinical features.

Clinical attribute	Attribute type	Statistical test	*p*-value	q-value
TMB	Sample	Wilcoxon test	< 10^− 10^	< 10^− 10^
Sex	Patient	Chi-squared test	1.35E−05	3.78E−05
Mutation count	Sample	Wilcoxon test	< 10^− 10^	3.01E−10
MSIsensor score	Sample	Wilcoxon test	7.14E−05	1.90E−04
Tissue source site code	Sample	Chi-squared test	8.75E−05	2.23E−04
Subtype	Patient	Chi-squared test	< 10^− 10^	< 10^− 10^

### Immunohistochemical validation of GNGT1 expression

**Fig. 6:**
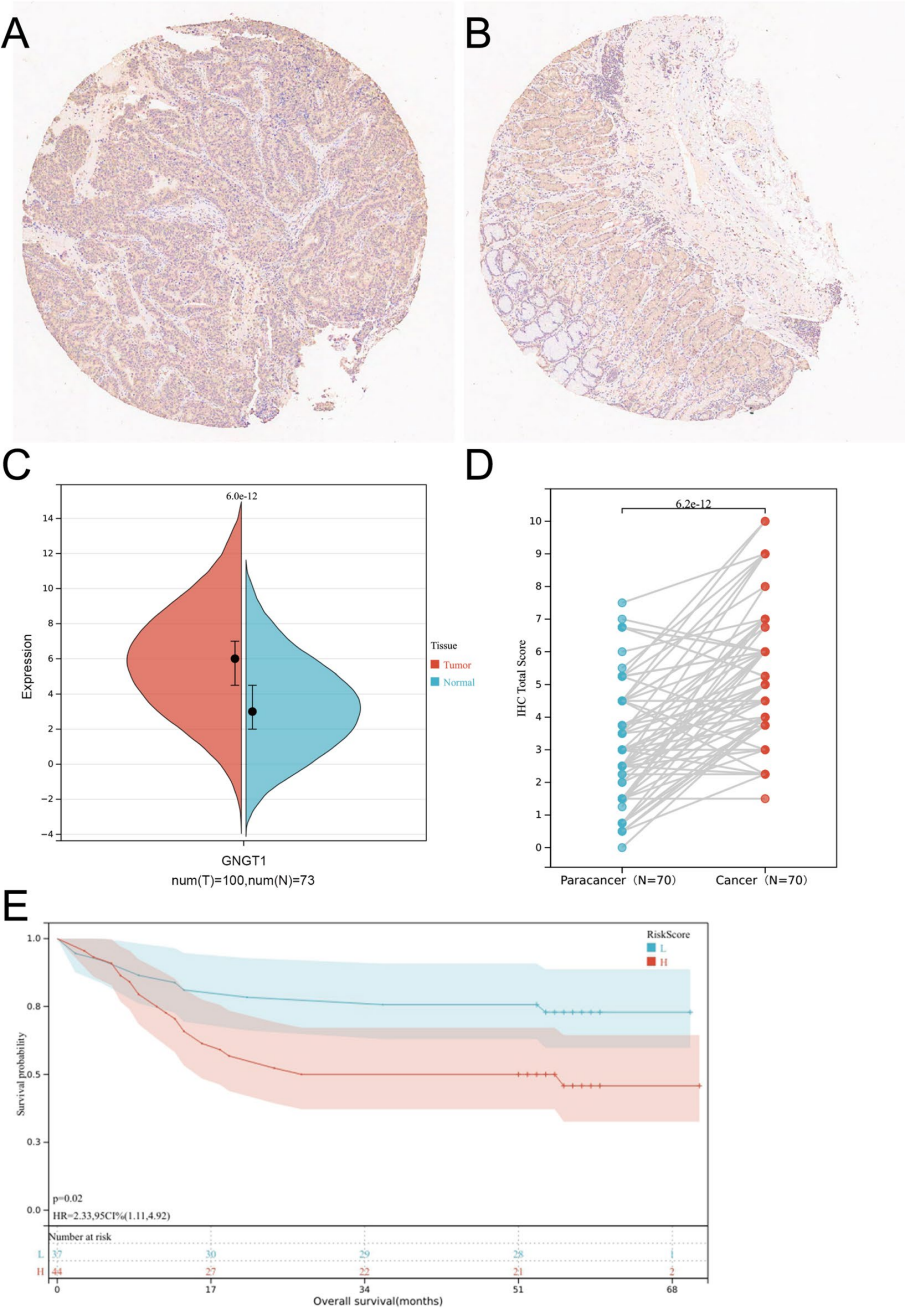
GNGT1 expression is upregulated in clinical samples from patients with GC and associated with shorter survival. (**A**) GNGT1 protein IHC staining in GC tissues, (**B**) GNGT1 protein IHC staining in adjacent normal tissues, and semiquantitative IHC analysis of GNGT1 protein in GC tissues (*n* = 100) and normal tissues (*n* = 73). (**D**) Immunohistochemical staining scores of GNGT1 expression in paired tumour and paracancerous normal tissues from 70 patients with STAD in tissue microarrays. (**E**) Patients with GC with high GNGT1 expression had a much lower survival rate than those with low GNGT1 expression.

Immunohistochemical methods were used to assess the expression of GNGT1 in 104 GC tissues and 76 adjacent normal tissues. Finally, 70 tissue pairs were included in the statistical analysis. The GNGT1 staining intensity in GC tissues significantly increased, according to the results of our semiquantitative analysis (Fig. 6A–D). According to the survival study, the OS time (*P* = 0.02, Fig. 6E) was longer for GC patients with lower GNGT1 protein levels. In accordance with our findings from the TCGA and GEO databases, we propose that an elevated GNGT1 protein level is a risk factor for a poor GC prognosis.

### Functional enrichment analysis

**Fig. 7:**
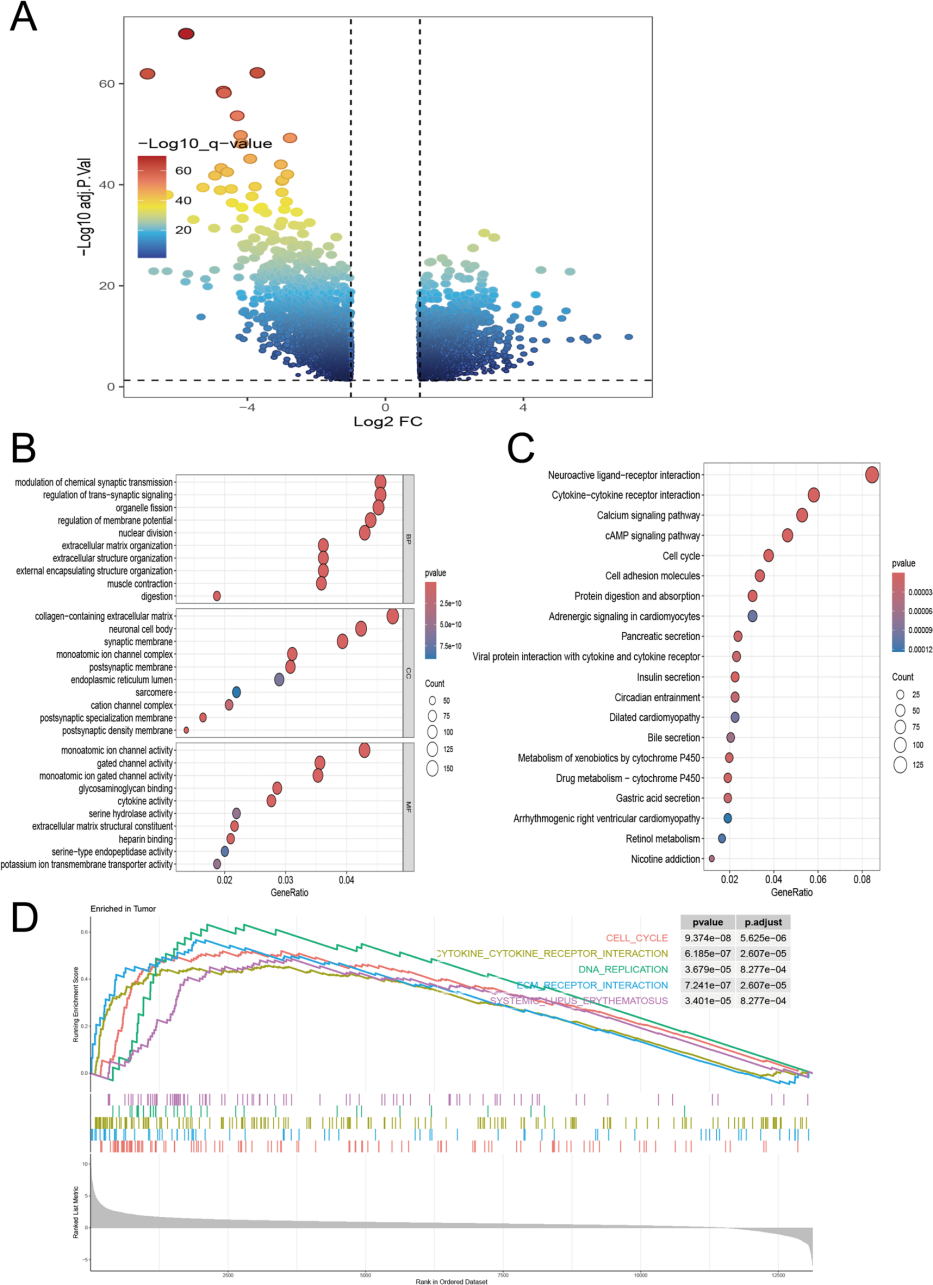
Visualization of 3508 DEGs. (**A**) Volcano plot of 3508 DEGs (the left dots indicate downregulated genes, and the right dots indicate upregulated genes), (**B**) GO enrichment analysis of 3508 DEGs, (**C**) KEGG enrichment analysis of 3508 DEGs, and (**D**) single-sample GSEA of GNGT1. ns, *p* > 0.05, *p < 0.05, **p < 0.01, ***p < 0.001.

To investigate the potential functions of the GNGT1 gene in GC, we performed DEG analysis on data from the TCGA dataset. A total of 3,508 DEGs, including 1,633 upregulated genes and 1875 downregulated genes, were identified and are depicted in a volcano plot (Fig. 7A). GO and KEGG pathway analyses were conducted on these DEGs. The GO analysis revealed that these genes were associated primarily with processes such as cell–cell signalling, biological adhesion, modulation of adenylate cyclase activity in the regulation of transsynaptic signalling, G protein-coupled receptor signalling, cell adhesion through plasma membrane adhesion molecules, the mitotic cell cycle, calcium ion transport, cytokine activity, G protein-coupled receptor binding and signalling receptor binding (Fig. 7B). Similarly, the KEGG pathway analysis revealed that the DEGs were involved mainly in the cAMP signalling pathway, cytokine–cytokine receptor interactions, the cell cycle, neuroactive ligand–receptor interactions, chemical carcinogenesis, the p53 signalling pathway, ECM–receptor interactions and CAMs^24^ (Fig. 7C). Furthermore, GSEA was performed to explore the pathogenic mechanisms associated with GNGT1. The analysis revealed that in GC patients with high GNGT1 expression, the top five enriched pathways were involved in cytokine–cytokine receptor interactions, DNA replication, the cell cycle, ECM–receptor interactions, and systemic lupus erythematosus (Fig. 7D). Notably, these five pathways were all positively enriched. Other differentially enriched pathways included melanoma, the P53 signalling pathway, the cancer pathway and pyrimidine metabolism.

### Relationship between GNGT1 mRNA levels and the tumour immune microenvironment

Tumour-infiltrating immune cells (TIICs) reflect the host antitumour immune response, which is crucial for the occurrence and spread of tumours. We used the TIMER2.0 and TISIDB datasets to explore the connections between GNGT1 expression levels and TIICs. According to TIMER2.0, GNGT1 expression was significantly negatively correlated with the proportions of 10 TIICs, such as NK cells, macrophages, monocytes, myeloid dendritic cells, neutrophils, CD4+ T cells, CD8+ T cells, B cells, memory B cells and naive B cells. GNGT1 expression and the proportion of Tregs were significantly positively correlated. Similar relationships between GNGT1 expression and TIIC abundances in GC were obtained via TISIDB. These results further support the notion that the level of GNGT1 may play a key role in the enrichment of TIICs in the TME.

**Fig. 8:**
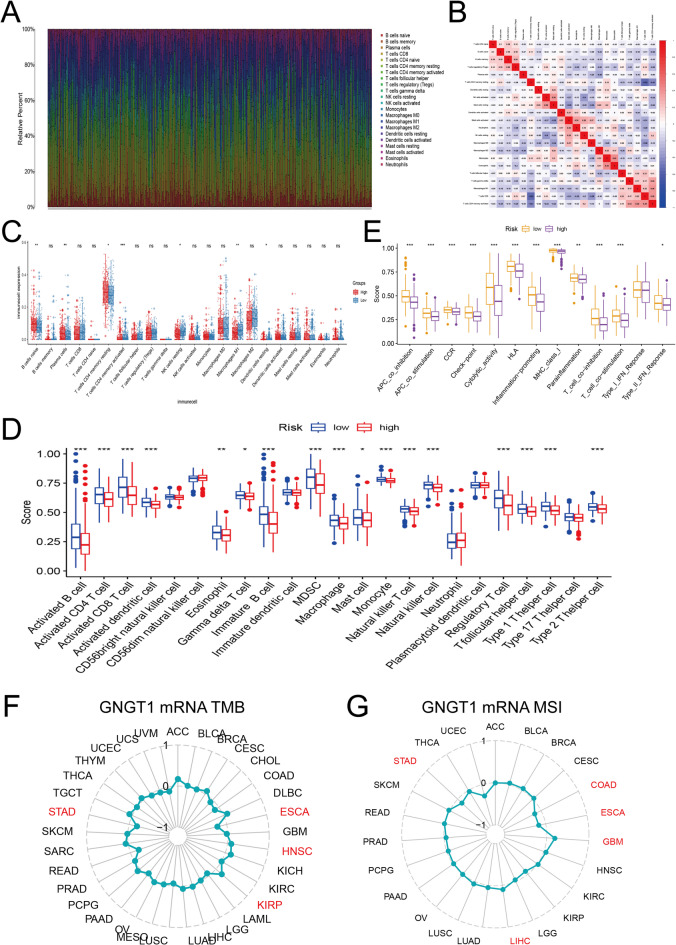
Analysis of the immune microenvironment in GC tissues from the TCGA database. (**A**) Relative proportions of 22 immune cell subtypes in 448 samples analysed by the CIBERSORT algorithm. (**B**) Correlation matrix of immune cell infiltration levels in GC tissue samples. (**C**) Differences in the proportions of 22 immune cell subtypes in GC tissues with high and low GNGT1 expression based on the CIBERSORT algorithm. (**D**) Differences in the proportions of 23 immune cell subtypes in GC tissues with high and low GNGT1 expression based on single-sample gene set enrichment analysis (ssGSEA). (**E**) Relationship between the GNGT1 expression profile and immune function. *p < 0.05, **p < 0.01, ***p < 0.001, ns, not significant (*p* > 0.05). Correlation of GNGT1 expression with the TMB (**F**) and MSI (**G**) in various cancer types.

CIBERSORT analysis was performed to assess immune cell infiltration in TCGA GC tissues, providing further insight into the association between GNGT1 expression levels and immune cell infiltration. We discovered changes in the proportions of 22 immune cell types in the GC tissue samples (Fig. 8A). We observed a substantial negative correlation (*r* = − 0.53) between the proportions of resting memory CD4 + T cells and CD8 + T cells and a significant positive correlation (*r* = 0.41) between the proportions of monocytes and eosinophils in the immune cell correlation matrix (Fig. 8B). Compared with tissues with lower GNGT1 expression, GC tissues with higher GNGT1 expression presented greater infiltration of initial B cells, memory CD4 + T cells and plasma cells but less infiltration of resting dendritic cells, resting NK cells, M1 macrophages and activated memory CD4 + T cells (Fig. 8C). Then, via R software, we conducted ssGSEA to evaluate the correlations between the proportions of 23 immune cells and GNGT1 expression in GC tissues from the TCGA. We discovered that increased GNGT1 expression was associated with reduced proportions of activated CD4+ T cells, activated B cells, activated CD8+ T cells, eosinophils, activated dendritic cells, γδ T cells, immature B cells, macrophages, monocytes, mast cells, natural killer T cells, regulatory T cells, myeloid-derived suppressor cells (MDSCs), follicular helper T cells, type 1 helper T cells, and type 2 helper T cells (Fig. 8D). In terms of immune function, increased GNGT1 expression was associated with decreased enrichment of the following gene sets: APC_co_inhibition, checkpoint, cytolytic_activity, APC_co_stimulation, CCR, HLA, MHC_class_I, T_cell_co-inhibition, inflammation-promoting, T_cell_co-stimulation, parainflammation and type_II_IFN_response function(Fig. 8E). In addition, GNGT1 expression was significantly correlated with MSI in STAD, COAD, LIHC, GBM and ESCA and with the TMB in STAD, ESCA, HNSC, and KIRP. In particular, GNGT1 expression was strongly negatively correlated with both the TMB and MSI in STAD (Fig. 8F,G). These findings strongly suggest that GNGT1 regulates the TME and is significantly associated with immunotherapy efficacy.

### Correlation analysis of GNGT1 with immune cell gene markers

In addition, we explored the potential relationships between GNGT1 expression and infiltrating immune cells in GC via the TIMER2.0 and GEPIA databases. There was a negative correlation between GNGT1 and several immune cell markers, including those of T cells, monocytes, CD8 + T cells, B cells, M2 macrophages, Th1-like cells, Tfh cells, Th2 cells, dendritic cells, naive T cells, resident memory T cells, exhausted T cells, effector memory T cells, central memory T cells, Tregs and TAMs. Specifically, GNGT1 expression was strongly associated with CD8A, HLA-DRA, and GZMA expression (Tables 2 and 3).

**Table 2:** Correlations of GNGT1 with immune cell gene markers in the TCGA-STAD cohort.

Description	Gene makers	TIMER2.0				GEPIA2	
		None		Purity			
		Cor	*p*	Cor	*p*	Cor	*p*
B cell	CD79A	− 0.126	**1.02E−02**	− 0.129	**1.17E−02**	− 0.13	**6.90E−03**
	CD19	− 0.101	**3.89E−02**	− 0.101	**4.96E−02**	− 0.042	3.80E−01
T Cell	CD2	− 0.213	**1.16E−05**	− 0.214	**2.71E−05**	− 0.18	**1.20E−04**
	CD3D	− 0.209	**1.71E−05**	− 0.212	**3.07E−05**	− 0.19	**4.50E−05**
	CD3E	− 0.203	**3.06E−05**	− 0.206	**5.42E−05**	− 0.18	**1.20E−04**
CD8 + Tcell	CD8A	− 0.224	**4.20E−06**	− 0.228	**7.54E−06**	− 0.23	**1.10E−06**
	CD8B	− 0.031	5.30E−01	− 0.03	5.58E−01	− 0.15	**1.70E−03**
Monocyte	CSF1R	− 0.225	**3.52E−06**	− 0.228	**7.05E−06**	− 0.22	**1.90E−06**
	CD86	− 0.196	**5.57E−05**	− 0.184	**3.08E−04**	− 0.15	**1.70E−03**
M1 Macrophage	NOS2	− 0.092	6.03E−02	− 0.105	**4.04E−02**	− 0.026	5.80E−01
	IRF5	− 0.012	8.10E−01	0.001	9.83E−01	0.028	5.50E−01
M2 Macrophage	CD163	− 0.174	**3.65E−04**	− 0.158	**2.07E−03**	− 0.077	1.00E−01
	MS4A4A	− 0.199	**4.34E−05**	− 0.187	**2.54E−04**	− 0.17	**2.10E−04**
	VSIG4	− 0.173	**3.90E−04**	− 0.158	**2.03E−03**	− 0.17	**4.70E−04**
Neutrophils	CEACAM8	0.197	**5.25E−05**	0.201	**8.00E−05**	0.17	**3.60E−04**
	ITGAM	− 0.149	**2.35E−03**	− 0.122	**1.72E−02**	− 0.1	**3.50E−02**
	CCR7	− 0.18	**2.37E−04**	− 0.183	**3.47E−04**	− 0.15	**1.20E−03**
Dendritic cell	HLA-DPA1	− 0.225	**3.72E−06**	− 0.221	**1.42E−05**	− 0.21	**1.00E−05**
	HLA-DPB1	− 0.236	**1.22E−06**	− 0.234	**4.15E−06**	− 0.22	**2.00E−06**
	HLA-DQB1	− 0.173	**3.98E−04**	− 0.16	**1.73E−03**	− 0.11	**1.80E−02**
	HLA-DRA	− 0.255	**1.38E−07**	− 0.25	**8.05E−07**	− 0.22	**2.40E−06**
	CD1C	− 0.149	**2.29E−03**	− 0.147	**4.19E−03**	− 0.19	**4.60E−05**
	ITGAX	− 0.195	**6.55E−05**	− 0.173	**7.07E−04**	− 0.082	8.60E−02
NKT	KIR2DL1	− 0.067	1.74E−01	− 0.08	1.20E−01	− 0.078	9.90E−02
	KIR2DL3	− 0.036	4.66E−01	− 0.044	3.90E−01	− 0.13	**7.40E−03**
	KIR2DL4	− 0.106	**3.06E−02**	− 0.082	1.10E−01	− 0.083	7.90E−02
	KIR2DS4	− 0.132	**6.88E−03**	− 0.156	**2.28E−03**	− 0.091	5.50E−02
	KIR3DL1	− 0.115	**1.93E−02**	− 0.117	**2.31E−02**	− 0.13	**5.60E−03**
	KIR3DL2	− 0.096	**4.98E−02**	− 0.106	**3.92E−02**	− 0.087	6.70E−02
TAM	CD68	− 0.098	**4.52E−02**	− 0.068	1.90E−01	− 0.073	1.20E−01
	IL10	− 0.164	**8.08E−04**	− 0.141	**5.95E−03**	− 0.16	**1.10E−03**
	CCL2	− 0.124	**1.17E−02**	− 0.105	**4.18E−02**	− 0.094	**4.90E−02**

**Table 3:** Correlation analysis between GNGT1 and gene markers of different types of T cells in TCGA-STAD.

Description	Gene makers	TIMER2.0				GEPIA2	
		None		Purity			
		Cor	*p*	Cor	*p*	Cor	*p*
Th1	TBX21	− 0.193	**7.78E−05**	− 0.198	**1.03E−04**	− 0.15	**1.20E−03**
Th1-like	IFNG	− 0.167	**1.31E−06**	− 0.168	**3.43E−06**	− 0.11	**2.40E−02**
	HAVCR2	− 0.209	**1.71E−05**	− 0.198	**1.08E−04**	− 0.14	**3.60E−03**
	CXCR3	− 0.197	**5.25E−05**	− 0.211	**3.42E−05**	− 0.15	**1.30E−03**
	CXCL13	− 0.194	**7.26E−05**	− 0.187	**2.45E−04**	− 0.15	**1.80E−03**
	CD4	− 0.228	**2.57E−06**	− 0.226	**8.54E−06**	− 0.18	**8.90E−05**
Tfh	IL21	− 0.168	**6.10E−04**	− 0.143	**5.15E−03**	− 0.15	**1.10E−03**
Th2	STAT5A	− 0.213	**1.18E−05**	− 0.194	**1.47E−04**	− 0.13	**7.40E−03**
Th17	STAT3	− 0.037	4.57E−01	− 0.022	6.75E−01	0.049	3.10E−01
Treg	CCR8	− 0.075	**3.09E−02**	− 0.071	5.21E−02	0.016	7.30E−01
Resting Treg T-cell	FOXP3	− 0.115	**1.90E−02**	− 0.117	**1.21E−03**	− 0.035	4.60E−01
	IL2RA	− 0.184	**1.60E−04**	− 0.169	**9.31E−04**	− 0.12	**9.30E−03**
Effector Treg T-cell	FOXP3	− 0.115	**1.90E−02**	− 0.117	**1.21E−03**	− 0.035	4.60E−01
	CTLA4	− 0.113	**2.08E−02**	− 0.1	5.08E−02	− 0.082	8.50E−02
	TNFRSF9	− 0.191	**8.89E−05**	− 0.194	**1.50E−04**	− 0.11	**1.60E−02**
Effector T-cell	FCGR3A	− 0.17	**5.07E−04**	− 0.16	**1.83E−03**	− 0.085	7.50E−02
	TNFRSF9	− 0.181	**8.89E−05**	− 0.194	**1.50E−04**	− 0.11	**1.60E−02**
Naive T-cell	CCR7	− 0.18	**2.37E−04**	− 0.183	**3.47E−04**	− 0.15	**1.20E−03**
	SELL	− 0.185	**1.47E−04**	− 0.169	**9.30E−04**	− 0.12	**9.70E−03**
Effector memory T-cell	DUSP4	− 0.142	**3.77E−03**	− 0.138	**7.22E−03**	− 0.064	1.80E−01
	GZMK	− 0.227	**2.91E−06**	− 0.234	**4.07E−06**	− 0.21	**6.30E−06**
	GZMA	− 0.247	**3.40E−07**	− 0.241	**2.06E−06**	− 0.23	**1.10E−06**
	PDCD1	− 0.173	**4.02E−04**	− 0.168	**1.00E−03**	− 0.16	**5.00E−04**
	IFNG	− 0.167	**1.31E−06**	− 0.168	**3.43E−06**	− 0.11	**2.40E−02**
Resident memory T-cell	CD69	− 0.228	**2.77E−06**	− 0.222	**1.34E−05**	− 0.21	**8.30E−06**
	CXCR6	− 0.196	**5.87E−05**	− 0.185	**2.82E−04**	− 0.19	**8.80E−05**
Exhausted T-cell	HAVCR2	− 0.209	**1.71E−05**	− 0.198	**1.08E−04**	− 0.14	**3.60E−03**
	LAG3	− 0.169	**5.55E−04**	− 0.168	**1.02E−03**	− 0.14	**3.40E−03**
	PDCD1	− 0.173	**4.02E−04**	− 0.168	**1.00E−03**	− 0.16	**5.00E−04**
	TIGIT	− 0.196	**3.33E−02**	− 0.195	**1.28E−04**	− 0.16	**3.60E−03**
	CXCL13	− 0.194	**5.97E−05**	− 0.187	**2.45E−04**	− 0.15	**3.40E−03**
	GZMB	− 0.171	**7.26E−05**	− 0.165	**1.23E−03**	− 0.12	**5.00E−04**
Central memory T-cell	CCR7	− 0.18	**2.37E−04**	− 0.183	**3.47E−04**	− 0.15	**3.60E−03**
	SELL	− 0.185	**1.47E−04**	− 0.169	**9.30E−04**	− 0.12	**3.40E−03**
	IL7R	− 0.2	**4.16E−05**	− 0.194	**1.49E−04**	− 0.16	**5.00E−04**

### Connection between GNGT1 expression and immune checkpoints

We investigated the relationships between GNGT1 expression in GC tissues and immune checkpoints to explore the role of GNGT1 in GC immunotherapy. GNGT1 expression was negatively correlated with the expression of 17 of the 30 immune checkpoint genes (VTCN1, TIGIT, SIGLEC9, SIGLEC7, SIGLEC15, PDCD1LG2, CYBB, HAVCR2, CD96, CD80, CD48, CD274, CD244, CD200, PDCD1 and LAG3) and positively correlated with the expression of two immune checkpoint genes (PVR and DIDO1). For some common ICs, we generated scatter plots to show the correlation with GNGT1 expression. In addition, immune checkpoint gene expression was compared between patients with high and low GNGT1 levels in the TCGA database. Three genes (VTCN1, PVR, and DIDO1) were upregulated in the high-GNGT1 group, and 13 genes (TIGIT, SIGLEC9, SIGLEC7, SIGLEC15, PDCD1LG2, HAVCR2, CYBB, CD96, CD48, CD274, CD244, PDCD1, and LAG3) were upregulated in the low-GNGT1 group. These results indicate that GNGT1 may be associated with the immune response in patients with STAD. Finally, we conducted analysis of somatic copy number alterations (SCNAs) in GNGT1. The infiltration levels of macrophages, dendritic cells, neutrophils, CD8 + T cells, CD4 + T cells and B cells were significantly correlated with GNGT1 SCNAs. These findings provide more evidence that GNGT1 levels may be critical to the infiltration of immune cells into the TME.

### Drug sensitivity analysis

**Fig. 9:**
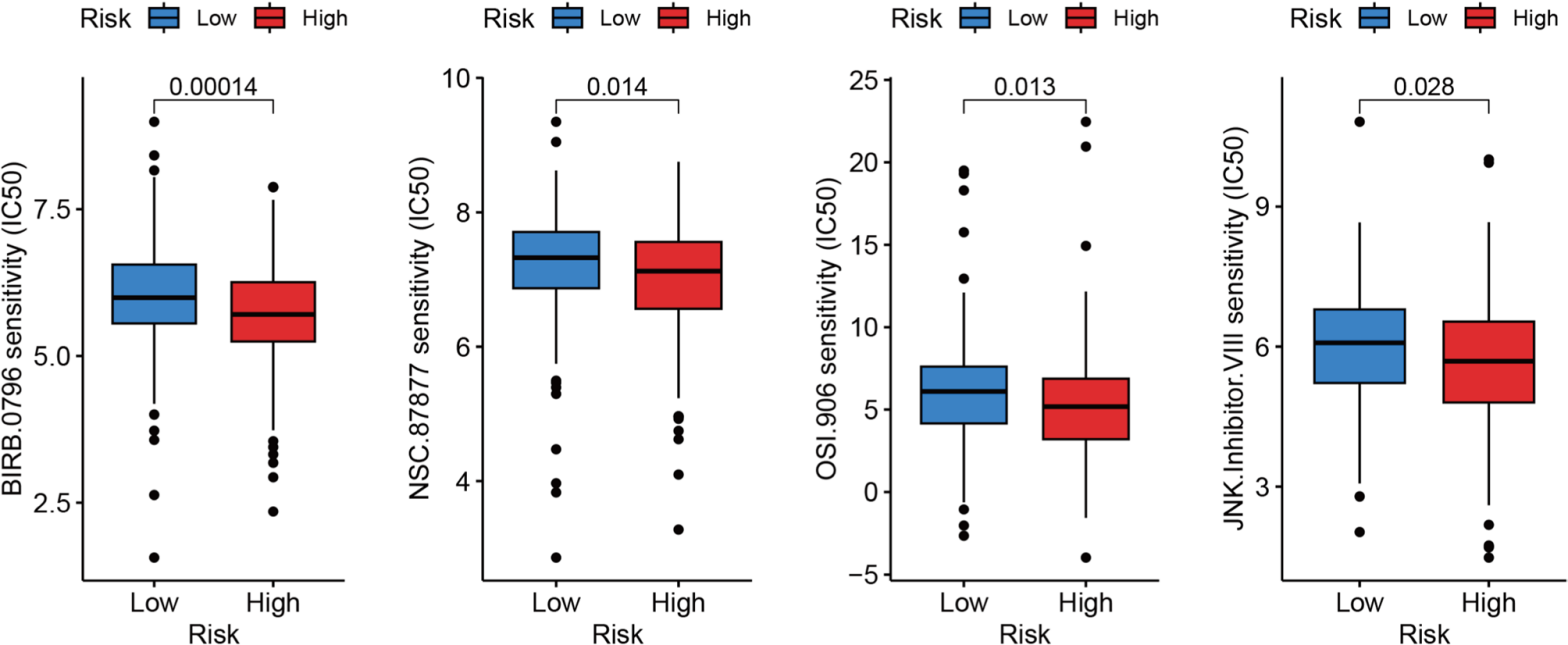
Differences in drug sensitivity between GC cell lines with low and high expression of GNGT1.

In our thorough analysis using the DepMap database, we assessed the pharmacological sensitivity of GC cell lines, categorizing them on the basis of GNGT1 gene expression level. Our investigation revealed a notable pattern: GC cell lines with high GNGT1 expression presented significantly increased sensitivity (log2FC<-1) to four distinct drugs (BIRB.0796, NSC.87877, OSI.906 and JNK.Inhibitor.VIII) (Fig. 9). Conversely, cell lines with reduced GNGT1 expression displayed a diminished response to the same set of therapeutic agents. Crucially, the difference in drug susceptibility between these two groups was statistically significant (*p* < 0.05).

## Discussion

Gene expression levels affect the onset and spread of GC^30^, indicating that some genes may be useful targets for diagnosis and treatment. As demonstrated by Chen and Lin et al.^16,17^, GNGT1 plays a critical role in GC. They showed that GNGT1 may be a major factor in the development of GC and that it is a useful biomarker for both diagnosis and treatment. However, rigorous bioinformatics investigations are needed to delve deeper into the role of GNGT1 in cancer progression to elucidate its particular significance in the development of GC.

The G protein family includes GNGT1, a gene that is responsible for encoding the γ subunit of transducin 1, a guanine nucleotide-binding protein (G protein) This subunit is associated with the RAS pathway and functions as a regulator or transducer in various transmembrane signalling systems^5^. A growing number of studies have recently shown that GNGT1 is abnormally expressed in various malignancies and may contribute to the initiation and spread of numerous cancers. Previous studies have demonstrated that GNGT1 helps reveal the molecular mechanisms underlying the occurrence and development of a variety of cancers, including invasive ductal carcinoma of the breast^10^, non-small cell lung cancer^11^, lung adenocarcinoma^12^, rectal cancer^13^, adrenocortical carcinoma^14^ and oesophageal squamous cell carcinoma^15^. GNGT1 was substantially found to be overexpressed in 14 cancer types relative to normal tissues, according to a pan-cancer characterization of its expression in the TCGA database (Fig. 2A). However, GNGT1 was downregulated in the tissues of both LGG and PRAD tumours, which might be related to these tumour types have distinct tumorigenic pathways. With respect to genetic mutations, GNGT1 genetic changes were found in approximately 1.8% of the GC patients. Additionally, we discovered that the majority of GNGT1 mutations were amplification mutations in GC, which may help to explain why they are highly expressed in GC. Furthermore, we conducted somatic mutation analysis in the TCGA database with a focus on GNGT1-related genes. The 30 genes with the highest rates of mutation were identified (Fig. 5D). The 5 genes with the highest frequency of mutations were ARID1A, LRP1B, MUC16, TP53, and TTN. Among the 431 samples, 397 (92.11%) had mutations, with missense mutations accounting for the majority. It was proposed that TTN mutations could predict a patient’s response to immunotherapy and that the TTN gene might be a possible therapeutic biomarker for GC^31^. In addition, TP53, LRP1B, and ARID1A were consistently the most frequently altered genes in all the populations^32^. Reduced ARID1A mRNA expression and elevated TP53 expression are significantly associated with advanced GC^33^. Similarly, LRP1B has been associated with a poor prognosis in patients with diffuse gastric carcinoma^34^. The MUC16 mutation status is associated with the TMB, MSI, survival and lymph node metastasis in GC patients^35^. In summary, these results suggest that the mutation status of TP53, TTN, MUC16, LRP1B and ARID1A is associated with GNGT1 expression, which provides clues for in-depth mechanistic studies and the development of targeted therapies.

According to data from several databases, GNGT1 expression is generally high in individuals with poor clinicopathologic features, and it is increased in numerous malignancies, including GC. In line with other GNG family member genes in cancer, GNGT1 mRNA was highly expressed in GC tissues. According to previous studies, GNG4 can stimulate colon cancer cell invasion, migration, and proliferation^36^ and is significantly expressed in colon adenocarcinoma cells and tissues^37^. Gliomas overexpress the oncogene GNG5, which can prevent glioma cells from proliferating and migrating, resulting in a poor prognosis^38^. Multiple findings indicate that GNG12 not only is a biomarker for glioma subtypes but also may promote tumour progression and that GNG12, which targets miR-876-5p, promotes the malignant progression of gliomas by increasing PI3K/AKT signalling activity^39^. In addition, Kaplan-Meier survival curves revealed that both GNGT1 expression and GNGT1 mutation were associated with survival outcomes in GC patients. In line with the impact of GNGT1 on the prognosis of NSCLC^11^ and LUAD^12^, patients with GC with greater levels of GNGT1 had shorter OS. More importantly, IHC was used in this study to analyse the protein expression level of GNGT1 in 104 GC tissues and 76 paracancerous tissue samples. The findings were in agreement with those of the previously described bioinformatics study (Fig. 6A–E). These results strongly imply that GNGT1 may play a protumourigenic role and that poor outcomes in GC patients are closely linked to high GNGT1 expression. Overall, our research verified that GNGT1 is a reliable prognostic indicator for GC patients.

However, studies on the biological functions of GNGT1 are still limited. We constructed a GNGT1 gene coexpression network using the TCGA-STAD dataset and carried out GO and KEGG enrichment analyses to elucidate the hidden mechanisms underlying its aggressive growth trend. According to this study, GNGT1, which is crucial for the development of GC, had the highest correlation with the expression of MKI67, COL1A1, SOX9, and MAL in STAD. For stage II/III colon cancer with mismatch repair defects and microsatellite instability, MKI67 may be a useful biomarker for diagnosis and prognosis evaluation^40^. COL1A1 was significantly upregulated in breast, colorectal, and gastric cancers, thus supporting its oncogenic role^41^. Epithelial SOX9 drives gastric adenocarcinoma progression and metastasis by inducing M2 macrophage repolarization through the paracrine leukaemia inhibitory factor (LIF) and suppressing CD8 + T-cell function^42^. In addition, MAL proteins inhibit GC cell metastasis and invasion by interfering with STAT3 phosphorylation^43^. GO and KEGG enrichment analyses of genes coexpressed with GNGT1 revealed that many classical signalling pathways, such as the cell–cell signalling, adenylate cyclase-regulated G protein-coupled receptor signalling, cytokine–cytokine receptor interaction, neuroactive ligand–receptor interaction, chemical carcinogenesis, cell cycle, cAMP signalling, ECM–receptor interaction, cell adhesion molecule (cam) and p53 signalling pathways, among others, were enriched. In addition, we performed GSEA of the DEGs in GC tissues with low and high expression of GNGT1. Our study revealed that GNGT1-related genes were enriched not only in the cell cycle, cytokine-cytokine receptor interactions, DNA replication, ACE receptor interactions, and systemic lupus erythematosus but also in the P53 signalling pathway, cancer pathway, and pyrimidine metabolism. Previous studies have elucidated the mechanistic link between GNGT1 and the PI3K-Akt signalling pathway in the development and progression of invasive ductal breast cancer^10^. The present study reveals for the first time the potential correlations between GNGT1 and the cAMP signalling pathway, P53 signalling pathway, and adenylate cyclase-regulated G protein-coupled receptor signalling pathway in STAD. Although existing research indirectly suggests that these biological functions are related to immunosuppression^44^, cancer proliferation and metastasis^45–50^, their relationships with GNGT1 are not specified. Our study provides new information on the functional role of GNGT1 in GC tumorigenesis.

According to recent research, immune cell infiltration patterns vary among cancer types, and tumour-infiltrating lymphocytes (TILs) have an immunoregulatory function in the TME, which is intimately linked to the immune escape of tumour cells and affects tumour growth^51^. Additionally, there is ongoing study on the potential of immune cells that infiltrate tumours to stimulate tumour development and aid in immunosuppression by delivering signals that facilitate the survival and multiplication of tumour cells^52^. Thus, we explored the correlation between GNGT1 expression and immune cell infiltration in GC patients via the CIBERSORT method. Our findings indicated that the infiltration of resting dendritic cells, activated memory CD4 + T cells, M1 macrophages and resting NK cells was significantly reduced in GC tissues with elevated GNGT1 expression.

We subsequently performed ssGSEA using the R software, which revealed that increased GNGT1 expression was related to decreased infiltration of activated CD8+ T cells, activated CD4+ T cells, activated B cells, eosinophils, activated dendritic cells, γδ T cells, macrophages, immature B cells, monocytes, mast cells, myeloid-derived suppressor cells (MDSCs), natural killer T cells, regulatory T cells, follicular helper T cells, type 1 helper T cells and type 2 helper T cells. In terms of immune function, increased GNGT1 expression was associated with decreased enrichment of gene sets involved in the following immune functions: APC_co_stimulation, APC_co_inhibition, CCR, HLA, inflammation-promoting, checkpoint, MHC_class_I, type_II_IFN_response, cytolytic_activity, parainflammation, T_cell_co-stimulation and T_cell_co-inhibition function. These findings indicate that GNGT1 may play a key role in immune microenvironment regulation. In addition, we explored the relationships between GNGT1 expression and infiltrating immune cells in GC by analysing the TIMER2.0 and GEPIA2 databases. We discovered that GNGT1 expression was negatively correlated with the expression of numerous immune cell markers, such as those of T cells, monocytes, B cells, CD8 + T cells, M2 macrophages, Th1-like, Tfh, Th2, and naive T cells, dendritic cells, resident memory T cells, exhausted T cells, effector memory T cells, costimulatory T cells, Tregs and TAMs. Specifically, we found that GNGT1 expression was strongly negatively associated with CD8A, HLA-DRA and GZMA expression (Tables 2 and 3). Our findings indicate that B cells can play a key role in antitumour immunity by shaping T-cell responses, thereby promoting adaptive and innate immunity^53,54^. By identifying and destroying altered cells, T cells also contribute significantly to tumour surveillance, and T-cell-mediated adaptive immunity is also believed to be a crucial factor in antitumour immunity^55^. By directing arriving T-cell effector development and sending survival signals to infiltrating T cells and intratumoural dendritic cells (DCs) to improve local anticancer immunity^56^. The progression of tumours is significantly influenced by the M1/M2 macrophage pattern. In contrast to M2-polarized macrophages, which are generally believed to be related to immunosuppression, hypoxia induction, tumour cell proliferation, metastasis, and the regulation of angiogenesis and lymphovascular generation, M1 macrophages have historically been thought to be antitumour macrophages. Thus, imbalances in these macrophage types can influence cancer development and progression^57^. Specifically, lower percentages of T cells, dendritic cells, M1 macrophages and B cells infiltrated into GC tissues with high GNGT1 expression, suggesting that GNGT1 may promote GC progression by decreasing the infiltration of T cells, dendritic cells, M1 macrophages and B cells.

In previous studies, no direct link was found between GNGT1 and the abnormal activation of immune checkpoint molecules. Nevertheless, our analysis revealed that GNGT1 expression was significantly positively correlated with that of PVR and DIDO1 and significantly negatively correlated with the expression of 17 immune checkpoint genes, including VTCN1, TIGIT, SIGLEC9, and SIGLEC7. We further compared immune checkpoint gene expression between patients with low and high GNGT1 levels. The results revealed that three genes (VTCN1, PVR, DIDO1) were upregulated and expressed in the high GNGT1 group, and 13 genes (TIGIT, SIGLEC9, SIGLEC7, SIGLEC15, PDCD1LG2, HAVCR2, CYBB, CD96, CD48, CD274, CD244, PDCD1, LAG3) were upregulated in the low GNGT1 group. We found that GC patients with patients with high MSI are more likely to benefit from ICIs^58^ and that a higher TMB is linked to a better prognosis and clinical response to ICIs^59^. According to our research, GNGT1 may be related to the immunological response in patients with STAD. Furthermore, a strong negative correlation was found between GNGT1 expression and both TMB and MSI. This finding may help explain why poor outcomes in GC patients are closely linked to high GNGT1 expression. Additionally, we found that the majority of immune cells and immune cell markers in GC tissues were negatively correlated with GNGT1 expression. These findings indicate that GNGT1 plays a role in tumour immunomodulation and is expected to be a new target for GC immunotherapy. However, the exact mechanism of GNGT1 in the TME still requires in-depth study.

GNGT1 has great potential value in GC diagnosis and prognosis evaluation, so it is necessary to determine whether it can be used as a viable drug target. Nevertheless, the therapeutic potential of GNGT1 is currently poorly understood. To determine which medication is likely to be more successful in treating individuals with GC who have high GNGT1 expression, we used the DepMap database. This study revealed that BIRB.0796, NSC.87,877, OSI.906 and JNK.Inhibitor.VIII have potential inhibitory effects on GC cells. BIRB.0796, a potent p38MAPK inhibitor^60^, reduces the synthesis of the pleiotropic cytokine oncostatin M (OSM)-induced vascular endothelial growth factor (VEGF)^61^, suggesting its therapeutic potential in GC. NSC-87,877 has been shown to potently inhibit growth and induce apoptosis in neuroblastoma cell lines, leading to reduced tumour growth and increased p53 and p38 activity^62^. However, because various cancer types behave differently, more research is necessary to confirm their effectiveness against stomach cancer cells. In addition, OSI-906, an insulin-like growth factor receptor-1 inhibitor, has been shown to inhibit the progression of GC^63^, ovarian clear cell carcinoma^64^ and colorectal cancer^65^. Notably, the role of JNK.Inhibitor.VIII in cancer therapy has not been published, and more studies are needed to investigate its potential applications. There is no proof that GNGT1 is directly involved in the aforementioned pharmacological mechanisms. Nonetheless, our novel research points to a possible synergy between GNGT1 targeting and treatment with these drugs.

Overall, our research highlights the role of GNGT1 in GC and clarifies its connection with the immune microenvironment, immune cell gene markers, immune checkpoints, and drug sensitivity. Our novel findings in these areas contribute significantly to the exploration of GNGT1. However, several limitations must be acknowledged. First, the data for our analyses were obtained from public databases, which means that we had no control over factors such as geographic origin or sample type. Consequently, the generalizability of our conclusions may be limited. Additionally, while we conducted a comprehensive analysis of GNGT1 mRNA expression, gene mutations, and TMB in GC and validated these bioinformatics findings with clinical cohort data, we were unable to fully elucidate the molecular mechanisms underlying the role of GNGT1 in GC cell growth and metastasis. Furthermore, the clinical validation cohort was limited to 104 GC patients, and data on immunotherapy efficacy were not available, preventing us from assessing the potential value of GNGT1 predicting immunotherapy efficacy. Despite these limitations, our study provides valuable insights into potential biological processes and pathways. Future research, including additional experimental validation and more extensive clinical studies, is needed to further refine and expand upon these findings.

## Conclusion

To verify the importance of GNGT1 in GC, we utilized rigorous and scientifically robust methods for both experimental validation and data analysis. Additionally, we comprehensively investigated the correlations between GNGT1 expression and key factors, such as tumour immune microenvironment features, immune cell gene marker expression, immune checkpoint expression and drug sensitivity, on the basis of multigroup bioinformatics and clinical cohort data. Our study demonstrated that GNGT1 expression was significantly upregulated in GC and that high GNGT1 expression may be associated with adverse clinical characteristics and clinical outcomes and is a potential biomarker for GC diagnosis and prognosis evaluation. GNGT1 may contribute to the development of GC by suppressing the infiltration of T cells, dendritic cells, M1 macrophages and B cells. It plays a crucial role in regulating the immune microenvironment of GC and works in conjunction with immune checkpoints to influence immune responses in patients. Furthermore, high GNGT1 expression is correlated with sensitivity to four different drugs. Overall, GNGT1 is a good biomarker for GC diagnosis and prognosis evaluation. It may also alter the response to ICIs by influencing IC expression and immune cell infiltration in the TME and is expected to be a novel target for GC immunotherapy. Although GNGT1 might not represent a novel biomarker for GC, our research advances our knowledge of its biological function. Further biological studies on the relevance of GNGT1 in GC are necessary to validate our current findings.

## Electronic supplementary material

Below is the link to the electronic supplementary material.

Supplementary Material 1

Supplementary Material 2

Supplementary Material 3

## Data Availability

The original contributions presented in the study are included in the article/supplementary material, further inquiries can be directed to the corresponding author.
