# Supplementary figures and images for: GNGT1 is a potential prognostic and immunologic biomarker in gastric cancer
Source: Sci Rep. 2025 Jul 1;15:21149. doi: 10.1038/s41598-025-08297-4 (PMC12217126; doi:10.1038/s41598-025-08297-4)

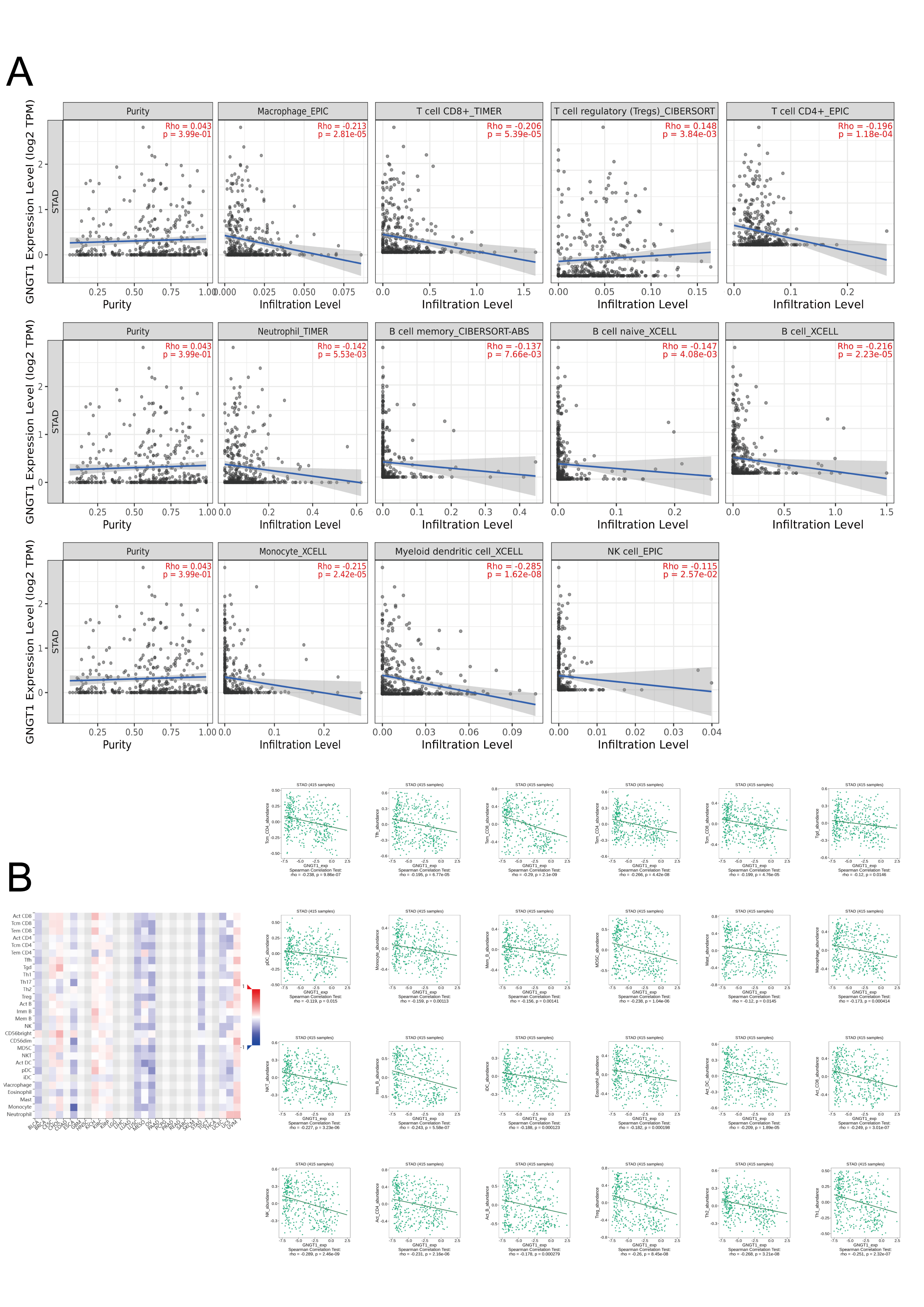

Supplement: Supplementary file 1 — Supplementary Material 1 [file 41598_2025_8297_MOESM1_ESM.tif]

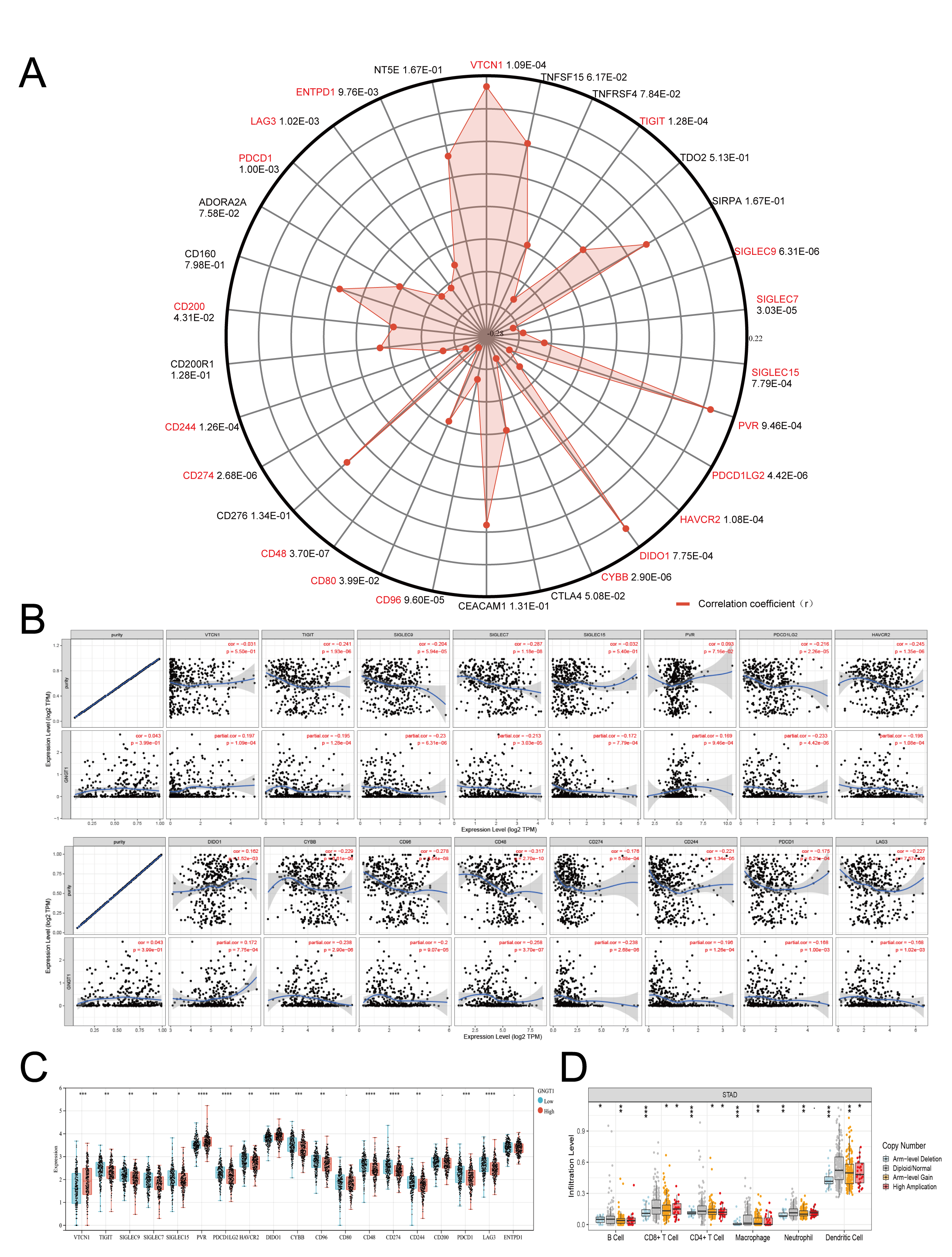

Supplement: Supplementary file 2 — Supplementary Material 2 [file 41598_2025_8297_MOESM2_ESM.tif]
