# Supplementary material for: GNGT1 is a potential prognostic and immunologic biomarker in gastric cancer
Source: Sci Rep. 2025 Jul 1;15:21149. doi: 10.1038/s41598-025-08297-4 (PMC12217126; doi:10.1038/s41598-025-08297-4)
Supplement: Supplementary file 3 — Supplementary Material 3 [file 41598_2025_8297_MOESM3_ESM.docx]

Supplementary Fig. 1. Associations between GNGT1 expression and TIICs. (A) Correlations between GNGT1 expression and 11 TIICs via TIMER2.0. (B) Correlations between GNGT1 expression and 24 TIICs via TISIDB.

Supplementary Fig. 2. Associations between GNGT1 and IC expression in GC. (A) Relationships between GNGT1 mRNA levels and IC expression. (B) Relationships between GNGT1 expression and VTCN1, TIGIT, SIGLEC9, SIGLEC7, SIGLEC15, PVR, PDCD1LG2, HAVCR2, DIDO1, CYBB, CD96, CD48, CD274, CD244, PDCD1 and LAG3 expression.(C) Differences in immune checkpoint-related gene expression between GNGT1 high and low expression groups. *P < 0.05; **P < 0.01; ***P < 0.001; ****P <0.0001. (D) Correlations between GNGT1 SCNAs and infiltration levels of dendritic cells, neutrophils, CD8+ T cells, macrophages, CD4+ T cells and B cells were analysed by using the TIMER database.
